# Organogenesis at the Shoot Apical Meristem

**DOI:** 10.3390/plants8010006

**Published:** 2018-12-28

**Authors:** Jan Traas

**Affiliations:** Laboratoire de Reproduction et Développement des Plantes, Université de Lyon, ENS de Lyon, UCBL, INRA, CNRS, 46 Allée d’Italie, 69364 Lyon CEDEX O7, France; Jan.Traas@ens-lyon.fr

**Keywords:** shoot meristem, morphogenesis, molecular regulation, cell wall, cytoskeleton

## Abstract

Lateral organ initiation at the shoot apical meristem involves complex changes in growth rates and directions, ultimately leading to the formation of leaves, stems and flowers. Extensive molecular analysis identifies auxin and downstream transcriptional regulation as major elements in this process. This molecular regulatory network must somehow interfere with the structural elements of the cell, in particular the cell wall, to induce specific morphogenetic events. The cell wall is composed of a network of rigid cellulose microfibrils embedded in a matrix composed of water, polysaccharides such as pectins and hemicelluloses, proteins, and ions. I will discuss here current views on how auxin dependent pathways modulate wall structure to set particular growth rates and growth directions. This involves complex feedbacks with both the cytoskeleton and the cell wall.

## 1. Introduction

Plants continuously make organs and tissues, thanks to the activity of meristems. Thus, the shoot meristems—at the tip of the stems and branches—initiate all the aerial parts, while the root meristems are responsible for the underground organs. The secondary meristems maintain the secondary growth of stems. I will focus here on lateral organ formation at the shoot apical meristem. Approaching the problem from a multi-scale perspective, we will discuss current evidence showing how molecular activity is translated into changes in geometry, while organs and tissues grow.

## 2. The Shoot Meristem: Molecular Regulation

The shoot meristem is a complex structure, divided into domains with specific functions [1]. At the tip of the meristematic dome is the so-called central zone, which contains the true stem cells. An intricate regulatory network determines the size and position of this population. At its heart is a signalling loop, which involves the transcription factor WUSCHEL (WUS), the receptor kinase (CLAVATA 1) CLV1, the receptor like protein CLV2 and the ligand CLV3 [2]. WUS is expressed in the so-called organizing centre at the base of the central zone, two or three cell layers deep. It activates CLV3 in the cells above and the ligand subsequently diffuses into the surrounding cells. Here, it interacts with the receptor complex CLV1/CLV2 to inhibit WUS. Many additional regulators have been identified, including partners of CLV1, components of diverse hormone signalling pathways, in particular cytokinin, as well as additional transcription factors active in other parts of the meristem. The meristem centre contains auxin, but there is evidence that proves it is not sensitive to the hormone [3]. Other non-elucidated interactions with meristem regulators such as SHOOTMERISTEMLESS also play a role. I will not discuss central zone regulation in further detail, but rather concentrate on what is happening at the periphery of the meristem when cells produced by the central zone enter differentiation. 

Cell growth and division push certain daughters of central zone cells to the periphery. These cells are in principle pluripotent and their daughters will be incorporated in organs or stem tissues. A major molecular signalling network involved in cell differentiation at the periphery is auxin (see e.g., [3,4]). The hormone is transported from cell-to-cell by membrane-localised transporters of the PIN family and accumulates at certain spots where it will launch the initiation of organ primordia. The importance of auxin transport in organ formation is illustrated by the phenotype of the *pin1* mutant in Arabidopsis [4]. This mutant is no longer able to transport auxin along its surface, and as a result forms naked inflorescence stems, unable to form flowers.

Auxin feeds into a complex regulatory molecular network. At the meristem, a range of transcriptional regulators is implicated in the early transduction cascade [3] that subsequently initiates further downstream events. In addition, cross talk with other signalling pathways, in particular that of cytokinin, is essential for correct organ initiation ([5,6,7,8] and references therein). Interestingly, many of the auxin-activated regulators are highly expressed at the periphery and only weakly in the meristem centre, although the auxin concentrations are high there [3]. This would suggest that auxin mainly acts in the peripheral zone. One of the main transcription factors activated directly by auxin is MONOPTEROS (MP) [9]. When MP is mutated, auxin can still accumulate, but organ formation is affected (see e.g., [4]). This is particularly striking at the inflorescence meristems, as the full knock-out *mp* mutant forms a naked, pin-like stem with very few or no flowers forming. An extensive analysis identified three other transcription factors as direct downstream targets of MP: AINTEGUMENTA (ANT), AINTEGUMENTA LIKE 6 (AIL6) and LEAFY (LFY) [10]. The triple *ant ail6 lfy* barely forms any organs, suggesting that all three genes are involved in organ outgrowth. Although this general model of auxin induced MP directly activating ANT/AIL6/LFY still stands, the triple mutant still produces some outgrowths that are still sensitive to auxin transport inhibitors, suggesting that other factors are involved [10].

Recent studies have revealed a more complex role of auxin in the more global coordination of meristem function. This involves transcription factors of the so-called APETALA 2 (AP2) family, DORNRÖSCHEN (DRN) and DORNRÖSCHEN-LIKE (DRNL) [11,12,13,14,15]. Both transcription factors are expressed in complementary domains at the SAM: DRN mainly at the central zone, and DRNL in the organ founder cells. Although this would suggest complementary roles, there is good evidence that both factors act synergistically in controlling CLV3 expression. Hereby, DRN directly binds the CLV3 promoter to positively regulate its expression. How DRNL affects CLV3 expression at a distance is not known at this stage [14]. Interestingly, DRN and DRNL, together with PUCHI, another transcription factor of the AP2 family, act synergistically in the control of floral organ number and even flower identity [12]. MP directly inhibits DRN at the peripheral zone. MP expression itself occurs along a gradient, with low expression at the meristem centre, thus allowing DRN to participate in the activation of CLV3 there [14]. In this manner, MP is also important in controlling the balance between meristem maintenance and organ formation at the periphery.

The regulators described above, only represent a very partial view of the molecular network. Other factors have been identified, and transcriptomic analysis has revealed that many genes are differentially regulated between the meristem centre and the periphery (e.g., [16]). The challenge for the future will be to produce a more complete, integrated model of the molecular network coordinating meristem function.

## 3. Translating Molecular Regulation into Changes in Geometry

So far, I have only considered the molecular regulation of meristem function. The next question is how this network of transcription factors and signalling molecules leads to the actual changes in shape we observe during organ outgrowth at the SAM. Growth is a physical process and the deformation of living tissues requires mechanical forces, which cause cells to grow at a certain rate and into a certain direction. We should therefore, not only look at morphogenesis from a geometrical point of view, but also consider the physical, structural components of the growing cells, in particular the extracellular matrix, called the cell wall. In the rapidly growing meristematic cells, these walls can be described as dense networks of cellulose fibres (microfibrils) cross-linked to a matrix that is largely composed of pectins and hemicelluloses (for reviews see: e.g., [17,18,19,20]. The matrix components can occur in different forms with different properties, defining their mechanical characteristics and capacity to bind to other wall elements.

The regulation of plant cell growth is closely linked to this cell wall structure ([18,19,20,21,22] and references therein). The cell walls are constantly under tension because of the internal turgor pressure. In addition, since the walls form a continuum linking the cells together, differences in growth rates between neighbouring cells can also influence the tensile forces acting on the individual walls. Together these forces form a tissue-wide stress field, causing the elastic deformation of the walls. According to widely accepted hypotheses, growth occurs when the cell walls yield to these forces and start to deform plastically. The yielding threshold depends on the degree of cross-linking between the wall components and can be modified, for instance, through the activity of wall-modifying enzymes. In the meristem, the major targets of wall-modifying enzymes are pectins and hemicelluloses [23]. The plastic deformation of the wall causes it to become thinner, which is compensated by synthesis and the insertion of new polymers. Whereas the overall growth rate largely depends on parameters like wall stiffness (the degree of cross-linking between the polymers) or wall synthesis, growth directions depend mostly on the orientation of the cellulose microfibrils, which restrict growth along their length. This orientation depends on the trajectories of the membrane bound cellulose synthases, which are guided by the microtubule cytoskeleton at the cell cortex [24,25].

In order to control morphogenesis, the molecular regulatory networks have to interfere with the local composition and texture of the cell wall. This process is conceptually simple, but in fact extremely complex and involves hundreds of wall-synthesizing and wall-modifying enzymes, often with redundant functions [26]. In principle, turgor pressure can also vary, but since little or nothing is known about its regulation at the shoot apex, it will not be further discussed here. In the following paragraphs, I will briefly summarize some of the current knowledge regarding the regulation of wall properties during growth at the shoot apical meristem.

## 4. Controlling Growth Rates at the Meristem

As indicated above, it is thought that growth rates are determined at the level of individual cells, largely by controlling wall stiffness and synthesis. Although we are only at the beginning of our understanding, there is strong evidence to suggest that local wall properties are very actively regulated during organ formation.

In an extensive analysis of over 150 enzymes involved in the synthesis of wall polymers, Yang and colleagues (2016) [27] found that most of them showed distinct patterns at the shoot meristem with a striking difference between the meristem proper and the young outgrowing organs. Armezzani et al. (2018) [23] also described strong differences in the expression of wall-modifying enzymes, in particular Expansins and XTHs, which in principle target hemicellulose and have the capacity to change wall stiffness.

How the expression of these genes is controlled is not precisely known, although a range of cell wall modifying enzymes have been identified as putative targets of meristem expressed transcription factors ([28,29]). Peaucelle and colleagues also identified potential roles of pectin modifications in organ outgrowth [30,31,32,33]. Pectin gels can be stiffer or looser depending on the degree of cross-linking of the individual polymers by Ca^2+^. Transgenic plants showing modified levels of specific forms of pectin show a dramatic reduction or increase in organ formation. In contrast to what these results might suggest, the intense activity of wall modifying genes does not lead to dramatic changes in wall mechanics. Measurements using atomic force microscopy have shown that wall stiffness is reduced during organ formation, but this remains within a limit of 20–50% at most [34].

## 5. Controlling Growth Directions at the Meristem

What about growth directions? Although differences in stiffness between individual walls can be involved [35] there is a general consensus that growth directions are mostly determined by the anisotropic properties of the cellulose network. If most microfibrils are aligned in one particular direction, they will restrict growth in that direction. As said above, microfibril orientation is regulated by the microtubule network, which guides the cellulose synthase complexes in the membrane. Accordingly, microtubule arrays are often (but not always) very precisely aligned perpendicular to the main growth direction [25]. How are these arrangements controlled? Since microtubule dynamics is not the main topic here, I will only give a very short overview, and highlight two general non-exclusive hypotheses—linked to the capacity of microtubules to self-organize into bundles. This depends in principle on a limited set of basic properties, such as polymerization/depolymerization, alignment (‘zippering’) and severing (cutting) [36], which involves an extensive set of associated proteins. The first hypothesis proposes cell geometry as an important organising factor [36,37,38,39]. Since microtubules and especially microtubule bundles are relatively stiff, they do not easily bend around the sharp cell corners in the small meristematic cells. In addition, the obstacles formed by these corners can affect microtubule stability and cause rapid depolymerisation. Therefore, cell geometry might play a significant role in microtubule organisation. This does not explain, however, why microtubules can show coherent alignment in neighbouring cells with sometimes very different shapes. We will consider the second hypothesis, which proposes that microtubules align along mechanical stresses [40] in somewhat more detail. The general idea here is that tissue-wide stress patterns generated by turgor pressure and differential growth (rapidly growing tissues ‘pulling’ on the more slowly growing ones) provide directional cues to the cytoskeleton. This generates a negative feedback loop, where the microtubules align the cellulose microfibrils along the main stress direction, thus causing the cells to resist the forces in that direction. Mechanical models show that in principle this should be sufficient to generate basic shapes such as cylindrical stems or dome shaped structures [40]. Evidence comes from work on the shoot apical meristem, where strong correlations between predicted stress patterns and microtubule alignments are found. Evidence also comes from hypocotyls and experiments where the stress patterns are perturbed, for instance using ablation or by applying external constraints [40,41]. This stress-based hypothesis for microtubule alignment provides a straightforward explanation for the coordinated behaviour of the structural elements in neighbouring cells. Although a mechanism involved in translating stress patterns into microtubule alignments has remained elusive, there are a number of interesting indications of how this could work. First of all, the direction of microtubule movements driven by motor proteins on artificial substrates in vitro is sensitive to stress, although the effects of this property in the living cell remains to be established [42]. In the context of morphogenesis at the shoot meristem, KATANIN (KTN), a protein involved in microtubule dynamics, stands out [43]. KTN is a so-called microtubule severing protein that destabilises local interactions between tubulin molecules. This supposedly promotes partial microtubule disassembly, efficient movement and, in rapidly growing plant cells, favours microtubule alignment. Interestingly, in mutants where KTN is impaired, the microtubule arrays are less organised and show a decreased capacity to align along predicted force patterns, even during mechanical perturbation [43]. Importantly, KTN directly interacts with RHO INTERACTING CRIB CONTAINING PROTEIN 1 (RIC1), which in turn interacts with RHO in PLANTS 6 (ROP6), thus potentially linking KTN function to cellular signalling [44]. Activation of the ROP pathway itself has been associated with auxin signal transduction, but how auxin is precisely perceived in this context remains a matter of debate [45].

How does the cytoskeleton behave at the shoot apical meristem? At the very tip of the meristem, microtubules mostly occur in isotropic (disorganised), dynamic networks. Towards the periphery they become highly anisotropic (organised, aligned) and the cells form tissue-wide microtubule arrangements surrounding the meristematic dome. This is particularly evident in organ boundaries [40,43]. As mentioned above, these supracellular arrangements correspond also qualitatively to the predicted stress pattern at the meristem surface. Important changes in these concentric patterns occur during organ formation. Soon after auxin accumulates, the microtubule arrays disorganise to become fully isotropic. In the context of the mechanical feedback hypothesis, this can be interpreted as the local inactivation of this feedback. The effect of auxin on the microtubules is thought to be a relatively direct effect, potentially involving ROP signalling [34].

Importantly, it seems to be sufficient to disorganise the microtubule arrays at the periphery to cause outgrowth, as drug treatments or mutations affecting microtubule alignments also lead to the formation of ectopic outgrowths or bulges on the meristem [34]. Mutations in KTN and treatment with the microtubule depolymerising drug, Oryzalin, even induce the formation of organs in the absence of auxin accumulation in the *pin1* mutant. Mechanical models have shown, that this shift to isotropic microfibril deposition could act in synergy with the relatively limited reduction in wall stiffness described above to induce rapid primordium outgrowth [23,34].

## 6. Not that Simple: Some Open Questions

**Figure 1:**
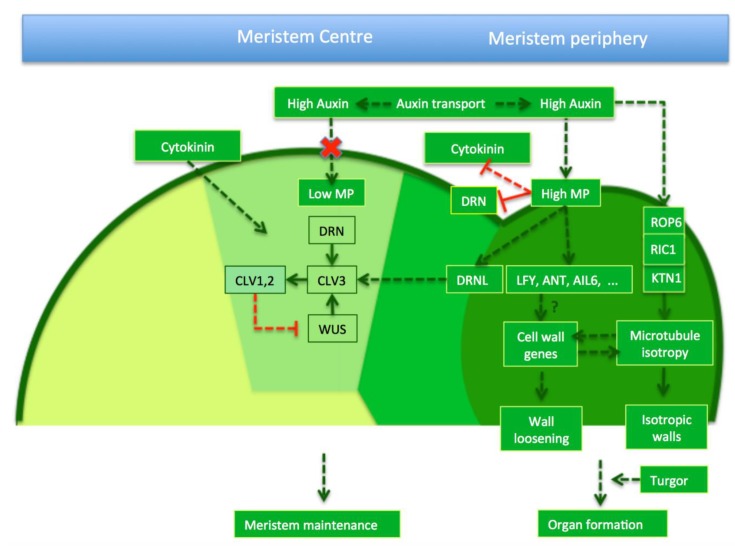
Molecular and cellular regulation of organ initiation at the periphery. Auxin transport generates auxin maxima at the meristem centre (light green area) and periphery (darker green), but since the centre is relatively insensitive to auxin (red cross), its effects seem to be limited and cytokinin driven meristem maintenance dominates. Auxin at the periphery causes wall loosening and cell isotropy. This involves both transcriptional and cellular responses. Depending on their wall properties, cells will then grow at particular rates and in particular directions, driven by turgor pressure. Dotted arrows represent indirect effects, solid lines direct, molecular relationships. Green arrows stand for positive control and red lines for inhibitions.

A scenario emerges, where auxin accumulation through transport activates downstream transcriptional regulation, leading to the activation of certain wall-modifying or synthesizing enzymes and a slight reduction in wall stiffness. In parallel, auxin—potentially via a KTN based signalling cascade—causes an inactivation of the mechanical feedback on microtubules (Figure 1). This leads to the disorganisation of the microtubule arrays and a switch to the isotropic deposition of cellulose microfibrils. Together these two effects of auxin act in synergy to cause the organ to bulge out, driven by turgor pressure.

**Figure 2:**
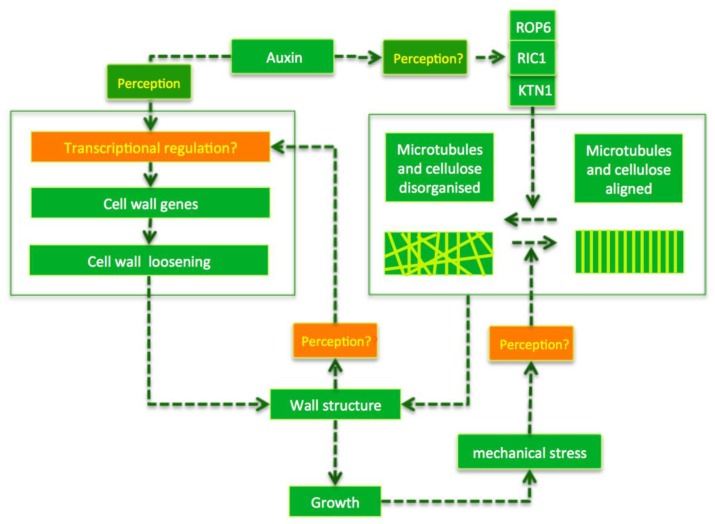
Some open questions. High auxin concentrations caused by auxin transport affects the cell wall structure in two manners during organ outgrowth: wall loosening (box at the left) and microtubule/microfibril organisation (pictured in the box on the right). Wall loosening involves transcriptional regulation. High auxin concentrations also promote a disorganisation of the microtubules, probably via a ROP/KTN based pathway (see also Figure 1), although this remains to be established. There is strong evidence that the cells perceive wall properties and mechanical stress and feed this information back to transcription and the cytoskeleton. Mechanical stress, for example, promotes microtubule alignment, while changes in wall anisotropy induce transcriptional responses. It is not known how wall structure and mechanical stress are perceived and transduced. Green arrows indicate positive regulation; orange boxes refer to the poorly understood processes that are discussed in the text.

This scenario leaves many questions open regarding the molecular players or the cellular signalling cascades involved. The precise changes in composition and mechanics of the cell wall during organ formation also remain almost a complete unknown. For the sake of simplicity, I have mainly discussed auxin here as an upstream regulator. However, there is strong evidence to support the idea that the localisation of auxin transporters is influenced by cell wall properties [46,47], pointing to the existence of some type of feedback towards signalling, which remains not understood at all. Here, I would like to highlight the following two points that are of particular interest (see Figure 2 for overview).

The first point concerns transcriptional regulation. As mentioned above, the presence of isotropic microtubule arrays at the meristem periphery is sufficient to cause organ outgrowth [34]. This outgrowth can even lead to the formation of flower-like structures in the absence of auxin transport as in *pin1 ktn* mutants. Importantly, this involves for example the transcriptional activation of cell wall modifying enzymes [23]. Therefore, a local switch to the isotropic deposition of cellulose fibres can also have effects on transcription and activate certain transcription factors required for flower formation and wall modification, even in the absence of high concentrations of auxin. In other words, there seems to be a feedback from the cytoskeleton to transcriptional regulation. How this works is completely unknown. In this context, it is worth noting that a range of membrane bound receptors have been associated with wall related signalling [48,49,50]. These receptors could potentially sense the mechanical status of the cell wall. This could even involve the direct binding of particular wall components such as pectins.

The second point of interest worth highlighting, concerns the mechanical feedback itself. As discussed, a number of components potentially involved in directional mechano-sensing have been identified. In addition, there is a strong correlation between microtubule alignment and predicted force patterns. To date, it is the only possible directional signal that coincides at least qualitatively with MT alignments at the meristem. Nevertheless, a negative feedback where microtubules align along the main force direction and cause the cells to resist to this direction leaves us with a fundamental contradiction. In principle, movement (strain) must be at the basis of force sensing. By reinforcing the wall along the main force direction, the microtubules also cause the cell to grow (i.e., to move) in a different direction. In other words, the main movement is no longer in the direction of the main force. Why is this movement not sensed by the microtubule arrays? How can they sense the main stress direction and not react to strain? The answer to this question is not yet known, but the evidence indicates that the effect of stress on microtubules must be indirect.
